# Prevalence of metabolic syndrome in China: An up-dated cross-sectional study

**DOI:** 10.1371/journal.pone.0196012

**Published:** 2018-04-18

**Authors:** Yu Lan, Zanlin Mai, Shiyu Zhou, Yang Liu, Shujue Li, Zhijian Zhao, Xiaolu Duan, Cao Cai, Tuo Deng, Wei Zhu, Wenqi Wu, Guohua Zeng

**Affiliations:** 1 Department of Urology, Minimally Invasive Surgery Center, The First Affiliated Hospital of Guangzhou Medical University, Guangdong Key Laboratory of Urology, Guangzhou, Guangdong, PR China; 2 The State Key Lab of Respiratory Disease, The Institute for Chemical Carcinogenesis, Guangzhou Medical University, Guangzhou, Guangdong, PR China; Sun Yat-sen University, CHINA

## Abstract

Metabolic syndrome (MS) is an increasing public health concern because of rapid lifestyle changes. Although there have been previous studies on the prevalence of MS in China, the prevalence may have changed with lifestyle changes over the last decade. To update this prevalence, we performed a cross-sectional survey among adults over 18 years old across China from May 2013 to July 2014. Participants underwent questionnaires and provided blood and urine samples for analysis. MS was defined according to the criteria of the China Diabetes Society. A total of 12570 individuals (45.2% men) with an average age of 48.8±15.3 (18–96) years were selected and invited to participate in the study. In total, 9310 (40.7% men) individuals completed the investigation, with a response rate of 74.1%. The prevalence of MS in China was 14.39% [95% confidence interval (CI): -3.75–32.53%], and the age-adjusted prevalence was 9.82% (95% CI: 9.03–10.61%; 7.78% in men and 6.76% in women; 7.39% in rural residents and 6.98% in urban residents). The highest prevalence occurred among adults aged 50–59 years (1.95%, 95% CI: 1.40–2.50%), and the lowest prevalence occurred among adults aged 40–49 years (0.74%, 95% CI: 0.38–1.10%); the prevalence was the highest in the south region and lowest in the east region (4.46% and 1.23%, respectively). The results of logistic regression analyses showed that age, urolithiasis, hyperuricemia, coronary artery disease, thiazide drugs intake, family history of diabetes and hypertension were all significantly associated with an increased risk of metabolic syndrome (OR>1). In addition, education, vitamin D intake and family history of urolithiasis are all protective factors (OR<1). Our results indicate that there was a high prevalence of MS in Chinese adults. Compared to the previous study 10 years ago, some preventive strategies have worked; however, further work on the prevention and treatment of MS remains necessary.

## Introduction

Metabolic syndrome (MS) is a complicated metabolic dysfunction disease comprised of a series of risk factors for angiocardiopathy, such as overweight, dyslipidemia, hypertension, and glucose intolerance [1]. MS is reported to be associated with an increased risk of many diseases, not only metabolic dysfunctions, such as type 2 diabetes (T2D) and cardiovascular disease (CVD), but also advanced colorectal polyps [2]. Thus, MS is a worldwide public health problem that causes direct and indirect economic burdens.

The prevalence of metabolic syndrome in China was 9.8% in 2005 [3] and 10.5% in 2009 [4]. However, this situation might have changed as a result of the rapid changes in lifestyle and dietary habits in China, which is the largest developing country in the world with a population of more than 1.3 billion. Therefore, it is important to survey the MS prevalence of China and to establish a preliminary understanding of the relevant risk or protective factors. The aim of the present study was to provide reliable and up-to-date information on the prevalence and associated factors of MS among adults over 18 years old in China.

## Subjects and methods

### Subjects

The subjects in this study were recruited from a cross-sectional survey that was performed to estimate the prevalence of kidney stones among adults aged ≥18 years from May 2013 to July 2014 in China [5]. The method of samples calculating could be seen in S1 File. In this study, we selected subjects via a multistage, stratified sampling method that was stratified according to the traditional seven geographical regions of China (south, south central, southwest, east, north, northeast, and northwest). The sampling process was divided into four tiers according to the size of the geographic area and the population of the regions. In the first two tiers, we selected provinces from these seven regions in a non-randomized way, and we selected cities and counties based on the degree of urbanization (cities vs villages). We selected streets (approximately 1 000–3 000 households) from the cities and villages (approximately 100–2 000 households) from the counties, and then we selected households from streets and villages for recruiting the subjects for randomization. All surveyed residents were 18 years or older and had lived in their current region for six months or longer.

This study was approved by the Ethics Committee of the First Affiliated Hospital of Guangzhou Medical University (S2 File). In addition, written informed consents were obtained from all participants, and all personal information was kept confidential.

### Data collection

All data were collected by the investigators of our research group in examination centers or community clinics of a participant’s residential area. To avoid selection bias, all the investigators had completed a same training program on the methodology and data acquisition procedures.

Height, weight and blood pressure (BP) of all participants were measured by systematically trained investigators. Body mass index (BMI) was calculated by the body weight in kilograms/ (body height in meters)^2^. The categories of BMI included underweight (BMI<18.5 kg/m^2^), standard weight (18.5–24.9 kg/m^2^), overweight (25–29.9 kg/m^2^), and obese (≥30 kg/m^2^). These definitions were based on the WHO guidelines [6]. BP was measured after fasting and being seated for at least 5 minutes. We used the mean BP value of three different measurements in our analysis.

The participants also responded to face-to-face questionnaires [5], and provided spot urine and blood samples for analysis. Information on demographic data, diet and lifestyle, and co-morbidities including hypertension and diabetes mellitus were recorded. Before using on study participants, questionnaire were tested about reliability by the method of test-retest reliability. 30 participants have been surveyed at an interval of 4 weeks with a kappa coefficient value of 0.71.

Urine samples were analyzed in the local hospitals. Blood samples were frozen and delivered to the central hospital of the province for analysis by automated analyzers. The standard blood analysis protocol included complete blood count, fasting serum glucose, creatinine, urea, uric acid, high-density lipoprotein, low-density lipoprotein, triglycerides, cholesterol, sodium, potassium, calcium and chloride. All laboratories of the study conformed to the standard requirements and obtained eligibility certification.

### Assessment criteria

The definition of MS in this study adheres to the CDS: to be diagnosed as MS, it must fulfill more than three of the following components:

1. BMI ≥ 25 kg/m^2^.
2. High serum glucose level: fasting plasma glucose (FPG) ≥6.1 mmol/L (110 mg/dl) and (or) 2 hPG≥7.8 mmol/L.
3. Blood pressure (BP): systolic blood pressure ≥140 mmHg and/or diastolic pressure ≥90 mmHg, and (or) have been diagnosed as hypertensive and undergone treatment.
4. Blood lipid disorders: fasting blood TG≥1.7 mmol/L (110 mg/dL) and (or) fasting blood HDL-C <0.9 mmol/L (35 mg/dL) (male) or <1.0 mmol/L (39 mg/dL) (female).

All investigators involved in this study had completed a training program on the methodology and data acquisition procedures. Each of them had a procedure manual that detailed the administration of the questionnaires, the operation of the urinary tract ultrasonography, how to collect measurements of blood pressure and anthropometric indexes, and how to collect and handle the blood specimens. All the collected data were registered and treated with Epidata software (version 3.1, Epidata Association, Denmark).

### Statistical analysis

Categorical variables were presented as proportions. Continuous variables are presented as the mean values with the corresponding 95% confidence interval (CI). Relevant characteristics were described and stratified according to the presence of MS. The prevalence of MS was stratified by gender, age, urban or rural residence, and geographic regions and is expressed as an adjusted rate with 95% confidence intervals (CIs). The age- and sex-adjusted prevalences of MS were estimated by the direct method using the known population distribution within China in 2010 [7]. Logistic regression analyses were used to test for risk factors with the odds of MS. With the use of backward elimination, only covariables that were significant (P<0.05) were calculated in the final model.

## Results

**Fig 1:**
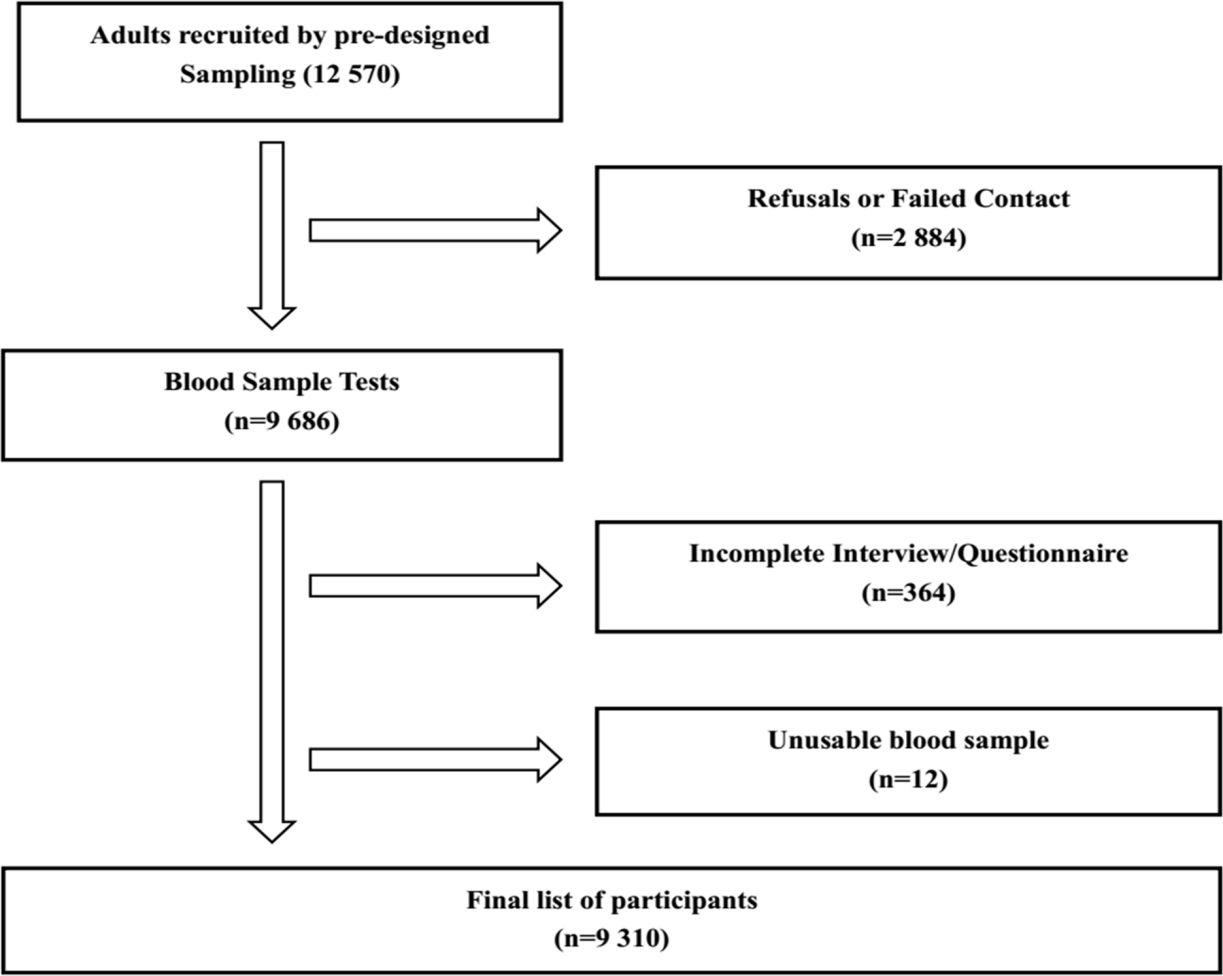
Flow diagram for recruiting subjects.

In total, 12 570 adults with an average age of 48.8±15.3 (18–96) years were randomly selected and invited to participate in this study, and 2884 participants were unavailable to participate due to contact failure, refusal, out-of-town work, and serious disability. Consequently, 9686 (77.1%) participants agreed to participate. Among these 9686 participants, 364 failed to complete the questionnaire and 12 had unusable blood samples. As a result, a total of 9310 individuals (3792 men and 5518 women) with an average age of 51.3±14.2 (18–96) years were included in the final analysis. The overall response rate was 74.1% (Fig 1).

The characteristics of the participants are shown in Table 1. The overall and sex-stratified prevalences of four metabolic dysfunctions and MS among adults in China are shown in Table 2. Among these 9310 participants, the overall crude/adjusted prevalences of the four components for MS were as follow: 32.35%/22.07% were overweight (BMI≥25 kg/m^2^), 28.22%/19.25% were hyperglycemic, 35.95%/24.52% had hypertension and 35.19%/24.00% had dyslipidemia, respectively. In total, 1340 participants (576 men and 764 women) were diagnosed with MS by duplication eliminating, and the overall prevalence of MS in China was 14.39% (95% CI: -3.75–32.53%). The overall age-adjusted prevalence of MS was 9.82% (95% CI: 9.03–10.61%). After standardizing the prevalence by gender, we found that the prevalence of MS was higher among men (7.78%, 95% CI: 6.93–8.63%) than women (6.76%, 95% CI: 6.10–7.42%). Furthermore, the prevalences of the four metabolic dysfunctions included in the diagnosis of MS were higher in men than in women in China (overweight: 17.43% of men and 15.22% of women; hyperglycemia: 15.24% of men and 13.25% of women; hypertension: 20.36% of men and 16.27% of women; and dyslipidemia: 19.31% of men and 16.33% of women) (Table 2).

**Table 1:** Characteristics of sample group by gender.

	Men (N = 3792) Mean (%), (95% CI)	Women (N = 5518) Mean (%), (95% CI)
Age (yr)	52.74 (52.27–53.21)	50.92 (50.55–51.30)
Height(cm)	166.27 (166.04–166.51)	156.53 (156.35–156.71)
Weight (kg)	66.10 (65.75–66.45)	57.91 (57.65–58.17)
BMI (kg/m2)	23.9 (23.8–24.0)	23.6 (23.5–23.7)
Glucose (mmol/L)	5.49 (5.44–5.55)	5.38 (5.34–5.41)
Cholesterol (mmol/L)	4.71 (4.67–4.74)	4.73 (4.70–4.76)
Triglycerides (mmol/L)	1.66 (1.62–1.70)	1.54 (1.51–1.57)
LDL-cholesterol (mmol/L)	2.60 (2.57–2.62)	2.62 (2.60–2.65)
HDL-cholesterol (mmol/L)	1.22 (1.21–1.23)	1.29 (1.28–1.30)
Systolic BP (mm Hg)	129.61 (128.97–130.25)	125.65 (125.10–126.21)
Diastolic BP (mm Hg)	81.74 (81.35–82.13)	78.92 (78.52–79.31)

Abbreviation: 95%CI: 95% Confidence Interval; BMI: Body Mass Index; LDL: Low Density Lipoprotein; HDL: High Density Lipoprotein; BP: Blood Pressure

**Table 2:** Prevalence of four metabolic dysfunctions among adults aged 18 years and older in China, 2013–2014.

	Overall (n = 9310)			Men (n = 3792)			Women (n = 5518)		
	n	Crude prevalence (%), (95%CI)	Adjusted prevalence (%), (95%CI)*	n	Crude prevalence (%), (95%CI)	Adjusted prevalence (%), (95%CI)*	n	Crude prevalence (%), (95%CI)	Adjusted prevalence (%), (95%CI)*
① BMI≥25.0	3012	32.35(30.68–34.02)	22.07(20.98–23.16)	1291	34.05(31.47–36.63)	17.43(29.00–33.38)	1721	31.19(14.93–47.45)	15.22(14.27–16.17)
② Hyperglycemia	2627	28.22(26.50–29.94)	19.25(18.21–20.29)	1129	29.77(27.10–32.44)	15.24(24.90–29.40)	1498	27.15(10.42–43.88)	13.25(12.36–14.14)
③ Hypertension	3347	35.95(34.32–37.58)	24.52(23.38–25.66)	1508	39.77(37.30–42.24)	20.36(31.18–35.48)	1839	33.33(17.33–49.33)	16.27(15.30–17.24)
④ Dyslipidemia	3276	35.19(33.55–36.83)	24(22.87–25.13)	1430	37.71(35.20–40.22)	19.31(31.30–35.60)	1846	33.45(17.46–49.44)	16.33(15.35–17.31)
①②③	774	8.31(6.37–10.25)	5.67(5.06–6.28)						
①②④	634	6.81(4.85–8.77)	4.65(4.09–5.21)						
①③④	753	8.09(6.14–10.04)	5.52(4.92–6.12)						
②③④	693	7.44(5.49–9.39)	5.08(4.50–5.66)						
①②③④	403	3.89(2.00–5.78)	2.95(2.50–3.40)						
MS	1340	14.39(12.51–16.27)	9.82(9.03–10.61)	576	15.19(12.26–18.12)	7.78(11.40–16.30)	764	13.85(-4.34–32.04)	6.76(6.10–7.42)

Abbreviations: CI: confidence interval; BMI: Body Mass Index; MS: metabolic syndrome

*: The prevalence was adjusted by the population 2010 in China.

**Fig 2:**
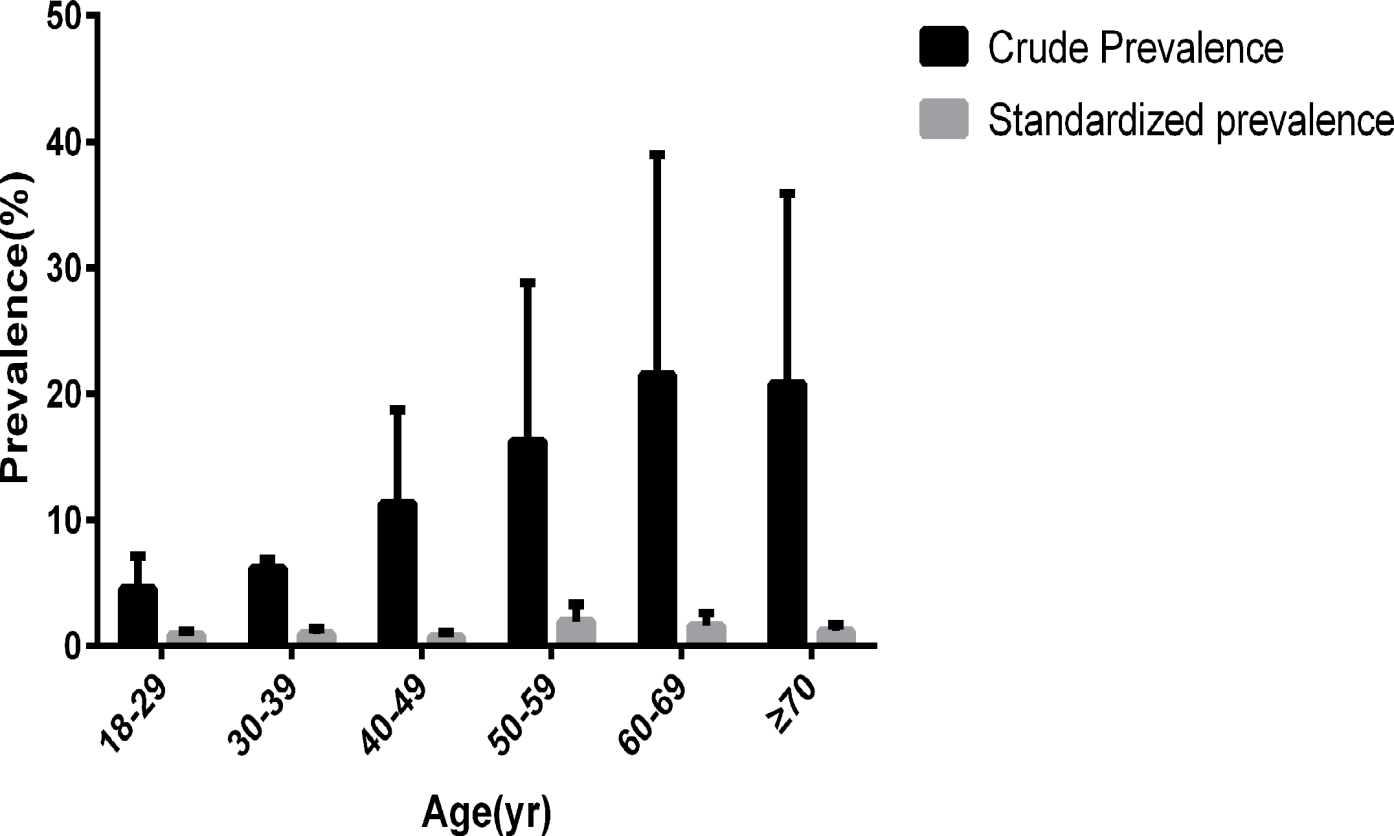
Prevalence of MS among Chinese adults aged 18 years and older.

After stratifying by 10-year age groups, the highest age-adjusted prevalence of MS was reported among adults aged 50–59 years (1.95%, 95% CI: 1.40–2.50%) and the lowest was among adults aged 40–49 years (0.74%, 95% CI: 0.38–1.10%) (Fig 2).

**Fig 3:**
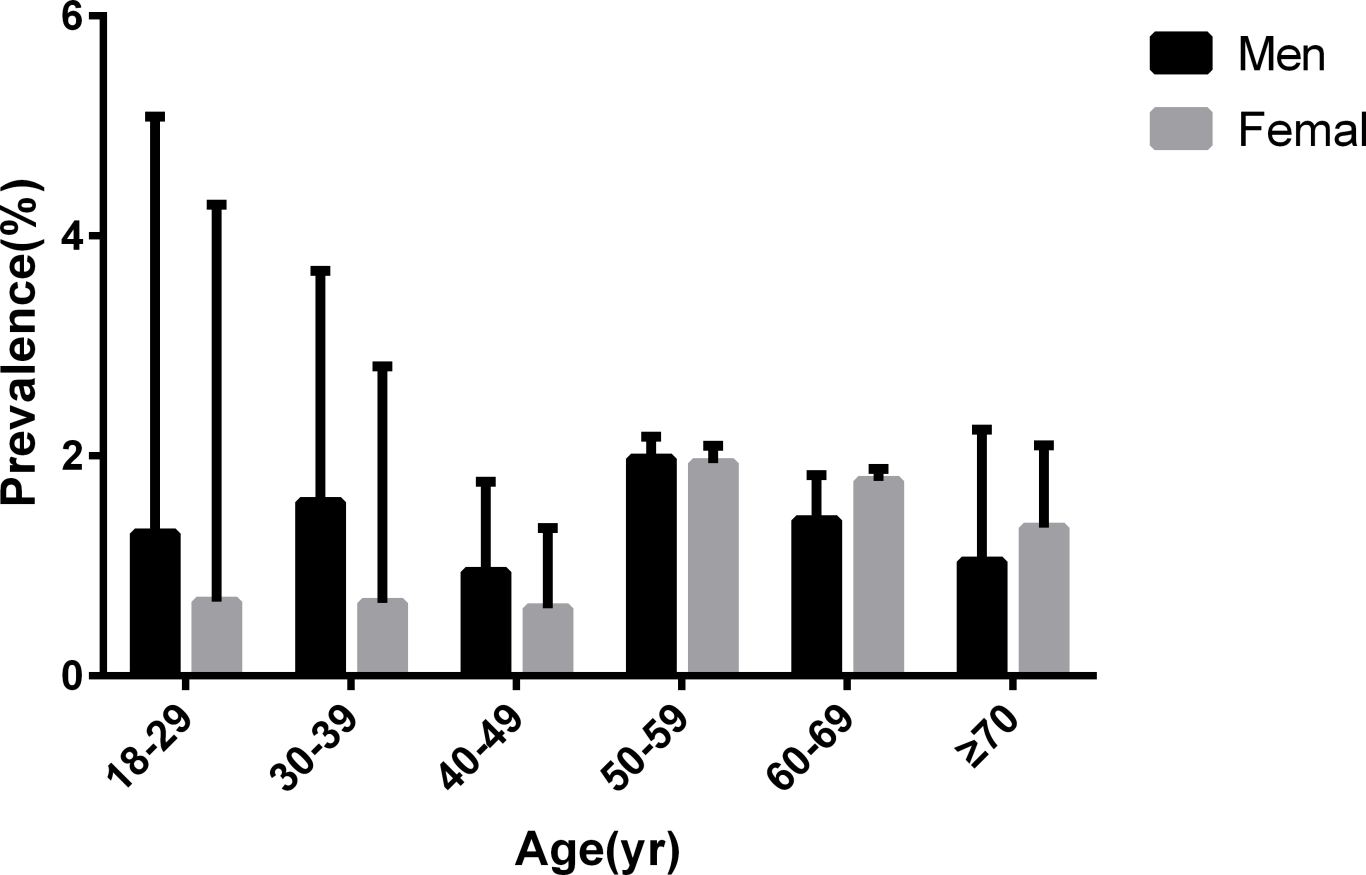
Age-specific prevalences of metabolic syndrome among Chinese adults aged 18 years and older.

After comparing the prevalence of MS in different genders in various age groups, we found that men had significantly higher levels of MS than women in subjects younger than 50 years old whereas both genders were equal between 50–59 years of age. However, the situation was reverse after 60 years of age (Fig 3).

Rural residents (7.39%, 95% CI: 6.65–8.12%) had a higher prevalence of MS than urban residents (6.98%, 95% CI: 6.23–7.73%) (Table 3).

**Table 3:** Prevalence of MS and 95% CI among urban and rural residents.

	Positive (No.)	Crude Prevalence (%), (95% CI)	Standardized prevalence (%), (95% CI)
Total			
Urban	616	13.89 (11.16–16.62)	6.98(6.23–7.73)
Rural	724	14.85 (12.26–17.44)	7.39(6.65–8.12)
Men			
Urban	285	6.42 (3.57–9.27)	1.65(1.06–2.25)
Rural	294	6.03 (3.31–8.75)	1.54(1.00–2.07)
Female			
Urban	331	7.46 (4.63–10.29)	1.83(1.32–2.34)
Rural	430	8.82 (6.14–11.50)	2.14(1.61–2.67)

Abbreviation: 95%CI: 95% Confidence Interval.

**Fig 4:**
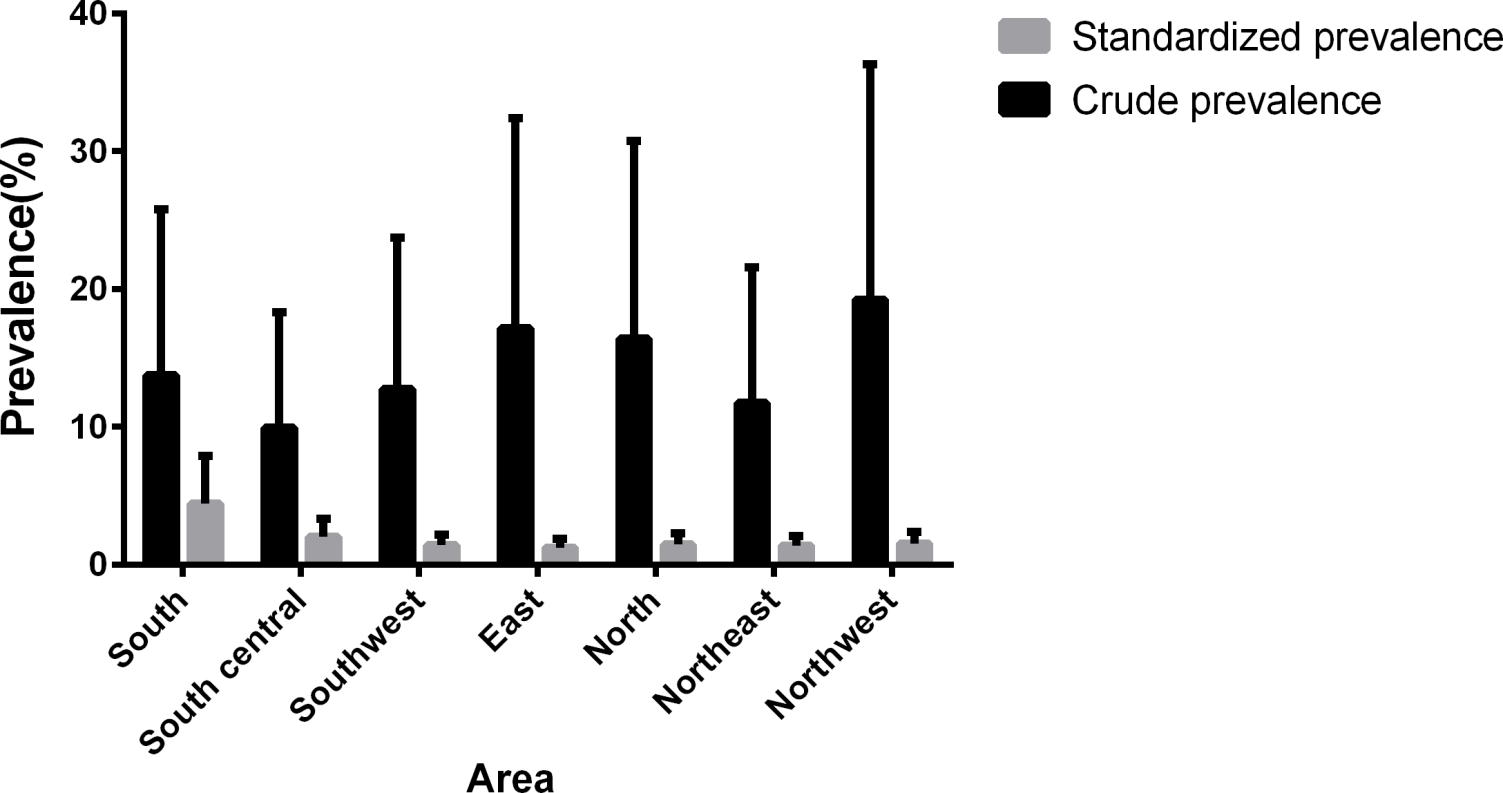
Prevalence of metabolic syndrome among the seven regions in China.

After comparing the prevalence of metabolic syndrome among the seven major areas in China, we found that the prevalence of MS was the highest in the south area (4.46%, 95% CI: 3.45–5.47%) and the lowest in the east area (1.23%, 95% CI: 0.65–1.81%) (Fig 4).

From the results of the logistic regression analyses (Table 4), the factors age, urolithiasis, hyperuricemia, coronary artery disease, thiazide drugs intake, family history of diabetes and hypertension were all significantly associated with an increased risk of metabolic syndrome (OR>1). In addition, education, vitamin D intake and family history of urolithiasis seemed to be protective factors (OR<1).

**Table 4:** Logistic regression analyses with the OR for metabolic syndrome.

Variable	OR (%), (95% CI)	P value
Age(18–29 years old)	-	< 0.001
Age(30–39 years old)	1.341(0.874,2.056)	0.179
Age(40–49 years old)	2.513(1.708,3.698)	< 0.001
Age(50–59 years old)	3.681(2.519,5.379)	< 0.001
Age(60–69 years old)	4.678(3.188,6.864)	< 0.001
Age(≥70 years old)	3.961(2.639,5.945)	< 0.001
Urolithiasis	1.283(1.027,1.603)	0.028
Hyperuricemia	2.467(2.086,2.917)	< 0.001
Education(illiteracy)	-	< 0.001
Education(1–6 years)	0.608(0.464,0.795)	< 0.001
Education(7–9 years)	0.498(0.398,0.624)	< 0.001
Education(10–12 years)	0.607(0.498,0.739)	< 0.001
Education(>13 years)	0.79(0.649,0.962)	0.019
CAD	1.587(1.238,2.035)	< 0.001
Vitamin D intake	0.647(0.408,1.027)	0.065
Thiazide drugs intake	2.493(1.371,4.533)	0.003
Family history of urolithiasis	0.615(0.452,0.837)	0.002
Family history of diabetes	1.325(1.075,1.634)	0.008
Family history of hypertension	1.589(1.374,1.838)	< 0.001

Abbreviation: OR: odds ratio; 95%CI: 95% confidence interval; CAD: Coronary artery disease.

## Discussion

In this current cross-sectional study, the results indicate that 130.9 million (or 14.39%) adults over 18 years old in China have metabolic syndrome. This estimated prevalence was similar to the data of 13.3% from a report in the *Chinese Journal of Preventive Medicine* [8] in 2002. Although it is much lower than the value of 23.7% in Americans [9], it is difficult to directly compare these two studies because of the different criteria used to diagnose MS. In our study, we found that there were nearly 294.2 million (or 32.35%) people who were overweight, 256.6 million (or 28.22%) people who had hyperglycemia or were taking hypoglycemic drugs, 326.9 million (or 35.95%) people who had hypertension or were taking antihypertensive drugs, and 319.9 million (or 35.19%) people who had dyslipidemia among those aged over 18 in China. Among those components of MS, hypertension had highest prevalence in men (20.36% in standardized prevalence) whereas dyslipidemia was the most common dysfunction in women (16.33% in standardized prevalence).

When analyzing the different age groups, we found that the prevalence of MS was lower among those under age 50 compared to older participants. This result may imply that the metabolic system weakens with aging. Additionally, a previous study showed that regular physical activity is beneficial for both blood lipids and blood pressure to reach an optimal level, and social labor primarily occurs among the youth and middle-aged, which will cause the above result [10]. In recent years, a strong awareness of avoiding a sedentary lifestyle, such as having a healthy recipe and keeping fit, was growing in the middle-aged in China. This may explain the result that those aged 40–49 had the lowest prevalence of MS.

The results from this study also show that men had a higher prevalence of metabolic syndrome than women, which is contrary to the result published by Gu [3]. This difference might be caused by the different criteria used. The criteria of metabolic syndrome used by Gu was the Adult Treatment Panel III (ATP III). The study by Gu found that women had a higher prevalence of abdominal obesity and lower HDL-cholesterol. After analyzing the prevalence of different genders in various age groups, we found that the situation can be divided into three associations. Interestingly, men had a clearly higher prevalence of MS than women before age 50, the difference between men and women was inconspicuous during ages 50–59, and the prevalence in women became higher than that in men after age 60. This association indicates that the changes of estrogen in women`s bodies may play a significant role in the mechanism of metabolic syndrome.

The standard prevalence of MS reported in our study was higher in the rural residents than in the urban residents, which differs from the previous result published by Gu. Therefore, the incidence of metabolic dysfunction has decreased in urban areas in the past decade. This decrease was associated with the growing consciousness of sports in cities, particularly after holding the Beijing Olympics in 2008. In addition, the increasing number of public fitness facilities after the introduction of the *Regulations of the National Fitness* by the Chinese government in 2009 has also contributed to the improved public health. Further, we found that rural women had a higher prevalence of MS than urban women, whereas rural men had a lower prevalence than urban men. This result might be caused by the fact that rural men performed much more physical work that benefits metabolic health than rural men.

When analyzing the relationship between metabolic syndrome and geographic factor in China, we learned that the prevalence of MS in Guangdong, which represents the south area, was the highest in China. The lowest prevalence existed in Shanghai, which represents the east area of China. Shanghai is one of the most economic and modernized cities in China. In the past decade, the importance of keeping health has been gradually realized in those modernized cities, which resulted in the increasing number of fitness centers and boom of the sports industry, simultaneously contributing to the low incidence of metabolic syndrome.

The results of the logistic regression analyses showed that education, having a family history of urolithiasis and vitamin D intake were protective factors of MS. However, there were several hazard factors such as aging, suffering from hyperuricemia or coronary artery disease (CAD) or urolithiasis, taking thiazide drugs, and having a family history of diabetes or hypertension that increased the risk of metabolic syndrome.

According to several previous studies, a high level of education was always associated with a low prevalence of metabolic dysfunction, such as diabetes [11] and cardiovascular risk factors including obesity, dyslipidemia, and hypertension [12]. Therefore, it makes sense that our study showed a significant inverse association between educational level and the prevalence of diabetes.

Those with a family history of urolithiasis could easily obtain information that physical inactivity and unhealthy diet may cause metabolic diseases. Thus, the initiative of keeping fit and having a healthy diet might be stronger in those individuals than the others, which make it possible for those individuals to have a lower occurrence of MS.

According to Kevin and his colleagues`survey of the National Health and Nutrition Examination Surveys 2003–2006 in US, Vitamin D intake and status are associated with lower prevalence of metabolic syndrome in adults. They found that those in the highest quartile of serum 25(OH) D had 60% lower odds for metabolic syndrome as compared to those in the lowest quartile, and those in the highest intake quartile had 28% lower odds for metabolic syndrome compared with the lowest vitamin D intake quartile (excluding supplements) [13].In addition, Ghanei L found that 98.4% of subjects with MetS and 88.3% without MetS had Vit. D deficiency and this difference was statistically significant (P = 0.005) [14]. Those were the same conclusion as we drew in this paper. Vitamin D intake is truly a protective factor of MS.

Many studies declared that hyperuricemia could also be treated as a diagnosed criterion of metabolic syndrome. According to a cross-sectional study performed by Chen JH and his colleagues [15], subjects with higher serum uric acid (sUA) levels had higher odds ratios (OR) for the occurrence of MS in the Chinese elderly aged over 65; thus, sUA levels could possibly be regarded as a potential tool for the early diagnosis of MS. In addition, a significant correlation was observed between sUA levels and MS; this association was stronger in premenopausal women than in postmenopausal women [16]. In our study, we also found that high sUA was a risk factor for MS, which supports the opinion that hyperuricemia ought to be considered a component of MS.

Those who took thiazide drugs generally had hypertension. As we know, diabetes and hypertension are genetic diseases to some extent, and both of these dysfunctions are components of MS. Thus, it is reasonable that having a family history of diabetes and hypertension is a risk factor for MS.

Urolithiasis was the most interesting risk factor for MS that we identified. According to a study published in European Urology, an increased prevalence of urolithiasis of over 75% was observed in those who were overweight and obese, and experts predicted that the economic cost in the treatment of urolithiasis due to obesity and diabetes was estimated to be approximately 1.24 billion US dollars per year by 2030 [17]. Additionally, a previous study noted that obesity was a significant contributing factor to urolithiasis [18]. The result that urolithiasis is a risk factor to MS, which we also found in this study, added proof to previous studies.

We performed this cross-sectional study using standard protocols and strict execution, detailed questionnaires, three blood pressure measurements, and standard laboratory tests for measuring glucose and lipids. Our findings provided an updated prevalence of metabolic syndrome in China compared to 2005. A high and increasing prevalence of MS was observed, which might be associated with the quickly developing economy and modernized lifestyle and diet. These findings indicate that metabolic syndrome has already become a major public health issue in China. Fortunately, we found that the prevalence of MS, which was previously higher in urban areas [3], has since decreased. This reduction may result from the stronger awareness of avoiding metabolic diseases in urban residents in the past decade. In addition, this change means that with more reasonable nationwide strategies, the prevention and treatment of metabolic syndrome could progress further.

## Limitations

There were some limitations in the present study. Firstly, the main results of this study were derived from a cross-sectional survey that was designed to observe the prevalence of urolithiasis in China. This survey lacked of data about waist circumference (WC), which prevented us from comparing various prevalences of MS by different diagnostic criterions, such as the US Third Report of the National Cholesterol Education Program (NCEP) Expert Panel on Detection, Evaluation and Treatment of High Blood Cholesterol in Adults (Adult Treatment Panel III) [19] and the International Diabetes Federation (IDF) criteria in 2005 [20]. Secondly, the response rate was only 74.1%. Because this cross-sectional survey required face-to-face interviews, some of the younger adults were unable to keep their appointments because of out-of-town work.

## Supporting information

S1 File The sample size calculation. (DOCX)

S2 File Ethical approval form. (DOCX)

S3 File Clinical studies checklist. (DOCX)

S4 File STROBE checklist. (DOCX)
