# Supplementary material for: Prevalence of metabolic syndrome in China: An up-dated cross-sectional study
Source: PLoS One. 2018 Apr 18;13(4):e0196012. doi: 10.1371/journal.pone.0196012 (PMC5906019; doi:10.1371/journal.pone.0196012)
Supplement: S1 File — (DOCX) [file pone.0196012.s001.docx]

**Sample size calculation**

In the cross-sectional survey of kidney stones in China, we estimated the prevalence of kidney stones about 6% according to previous regional study, an α of 0.05, a margin（$\delta$）of error of less than 2%, a sampling error of 10%, according to the formula as follow:$n=(\frac{Z_{1-\alpha/2}}{\delta})^{2}p(1-p)$, sample-size calculations suggested that 705 people would be needed in each site, with a predicted 20% refusal rate. And then a total of 9,870 peoples are required for 14 survey site in seven provinces.
