# Supplementary material for: Prevalence of metabolic syndrome in China: An up-dated cross-sectional study
Source: PLoS One. 2018 Apr 18;13(4):e0196012. doi: 10.1371/journal.pone.0196012 (PMC5906019; doi:10.1371/journal.pone.0196012)
Supplement: S2 File — (DOCX) [file pone.0196012.s002.docx]

**Ethical approval form**

| **Approval Form of Ethics Committee in the First Affiliated Hospital of Guangzhou Medical University** | | | | | | | |
| --- | --- | --- | --- | --- | --- | --- | --- |
| No.201231 | | | | | | | |
| Project | China National Epidemiological Study of Urolithiasis | | | | | | |
| Sponsor | Department of urology, The First Affiliated Hospital of Guangzhou Medical University | | | | | Head of this project | Guohua Zeng |
| Name | Gender | Job title | | Affiliation | | | Signature |
| Jinping Zhen | Men | Professor | | The First Affiliated Hospital of Guangzhou Medical University | | |  |
| Yuping Liu | Men | Professor | | The First Affiliated Hospital of Guangzhou Medical University | | |  |
| Ruliang Song | Men | Lawyer | | Nuochen Law Firm in Guangdong Province | | |  |
| Qiaoyuan Hong | Woman | Section chief | | Family planning office, Renming Street, Yuexiu District, Guangzhou | | |  |
| Qianming Yao | Men | Associate professor | | The First Affiliated Hospital of Guangzhou UniversityMedical | | |  |
| Guiqing Liu | Woman | Associate professor | | The First Affiliated Hospital of Guangzhou Medical University | | |  |
| Bing OuYang | Woman | Associate chief physician | | The First Affiliated Hospital of Guangzhou Medical University | | |  |
| Xinhua Xia | Men | Associate professor | | The First Affiliated Hospital of Guangzhou Medical University | | |  |
| Zhijian Tan | Man | Senior engineer | | The First Affiliated Hospital of Guangzhou Medical University | | |  |
| Voting results | Agreement 8 votes | Agreement after some necessary modification: 1 vote | | Disagreement: 0 vote | Stop the trail which was approved before:0 vote | | Members: 11; Be present: 9 Avoiding: 0; Give up: 0 |
| Submitted materials | List of materials: are presented in appendix | | | | | | |
| Date of review: 22/9/2012 | | | Review site: The meeting room in the 30th floor of The First Affiliated Hospital of Guangzhou Medical University | | | | |
| Contact address | Research service office, The First Affiliated Hospital of Guangzhou Medical University, 151#, Yuanjiang Road, Guangzhou, Guangdong Province | | | | | | |
| Tel. | 020-83062939 | | Fax:020-83177207 | | | Contact to: Dajia Yu | |

Comments of the Ethics Committee in the First Affiliated Hospital of Guangzhou Medical University:

The Ethics Committee has reviewed the project of "China National Epidemiological Study of Urolithiasis". This project is conducted by department of urology, the First Affiliated Hospital of the Guangzhou Medical University.

After reviewing and discussing the submitted materials(including ethics application form, ethics approval form, the protocol of this study, informed consent and so on ), the Ethics Committee of the First Affiliated Hospital of the Guangzhou Medical University concluded as following:

1. Agree to approve the scheme of the study, and the sponsor should carry out this study obeying the rules of the protocol strictly, and they should protect the rights of participants during the whole study.

2. If there are some updates of submitted materials, the sponsor should give them to the Ethics Committee in time. And when the study is carried out for a full year, the sponsor should present the research progress report to the Ethics committee. When the study is finished, please give the final report of the study to the Ethics committee.

Signature of chief: Jinping Zhen

Organization: the Ethics Committee in the First Affiliated Hospital of Guangzhou Medical University

Declaration: This Ethics Committee is an independent organization. The structure and the procedures of work are obeying the rules of GCP and the national laws. All the present members are in valid term.

**Approval Form of Ethics Committee in the First Affiliated Hospital of**

**Guangzhou Medical University (appendix)**

No.201231

| Project | China National Epidemiological Study of Urolithiasis | | |
| --- | --- | --- | --- |
| Sponsor | Department of urology, The First Affiliated Hospital of Guangzhou Medical University | Head of this project | Guohua Zeng |
| The lists of submitted materials and version number | | | |
| No. Name of document Version Date of version | | | |
| 1. Ethics application form | | | |
| 2. Ethics approval form | | | |
| 3. The protocol of this study | | | |
| 4. Informed consent (in Chinese) | | | |
|  | | | |
|  | | | |
|  | | | |
| Consider the previous comments of the study, the Ethics Committee has made a copy of the submitted materials about this study, and agree to approve the study of "China National Epidemiological Study of Urolithiasis".  The sponsor should carry out this study obeying the protocol strictly, and they should protect the rights of participants during the study. If there are some updates of submitted materials, the sponsor should give them to the Ethics Committee in time.  Signature of chief  Date | | | |

**
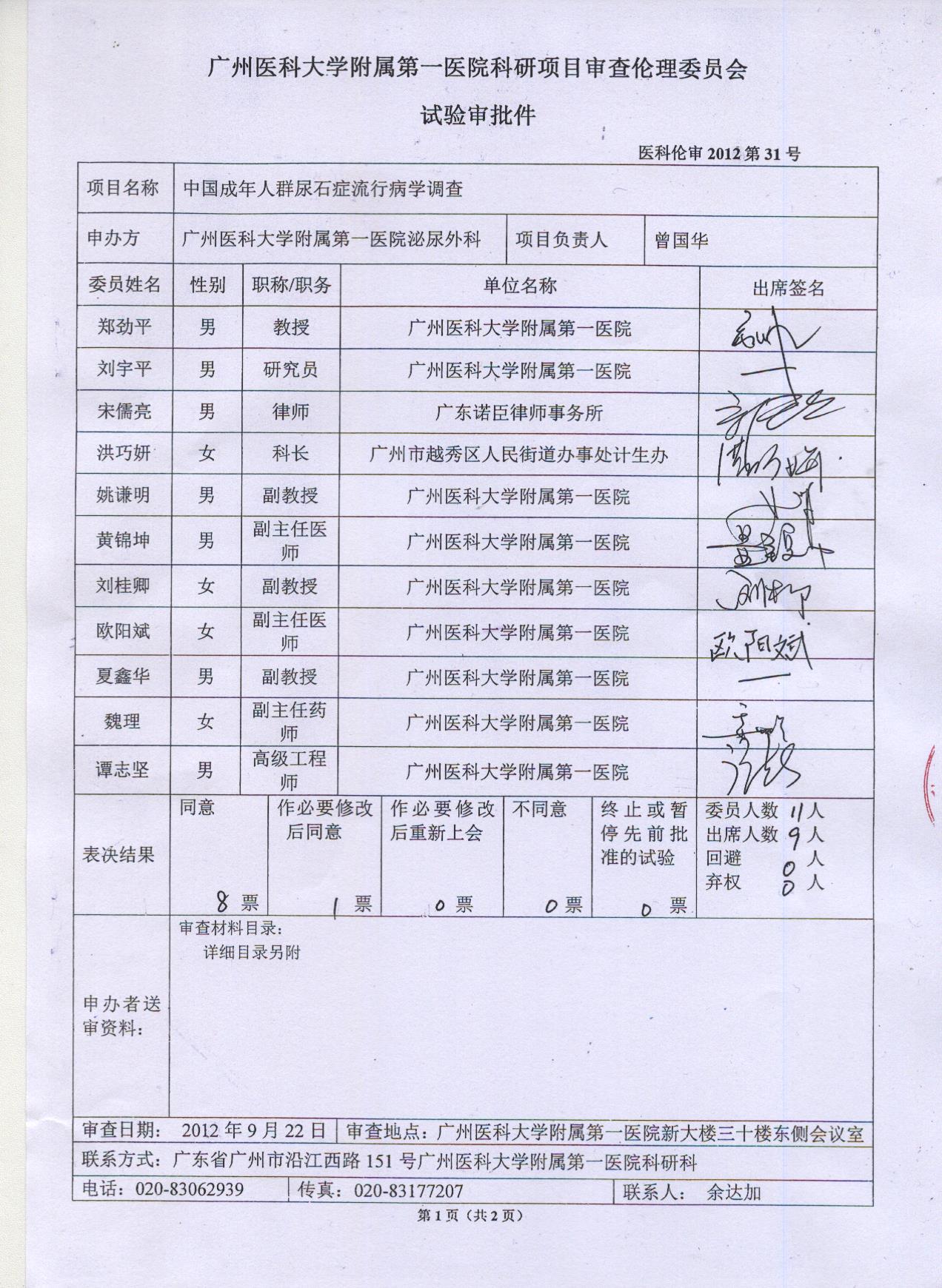
**

**
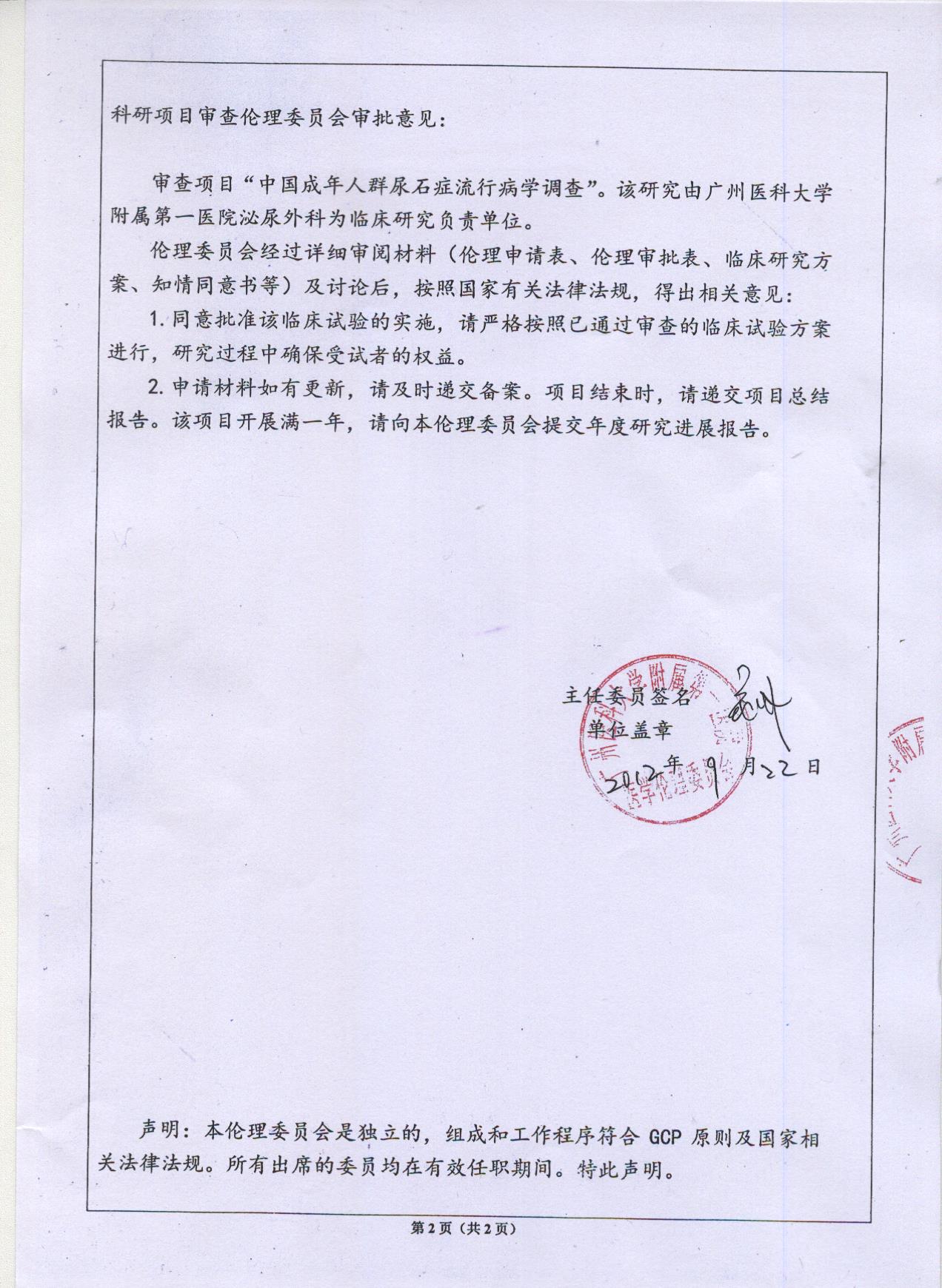
**

**
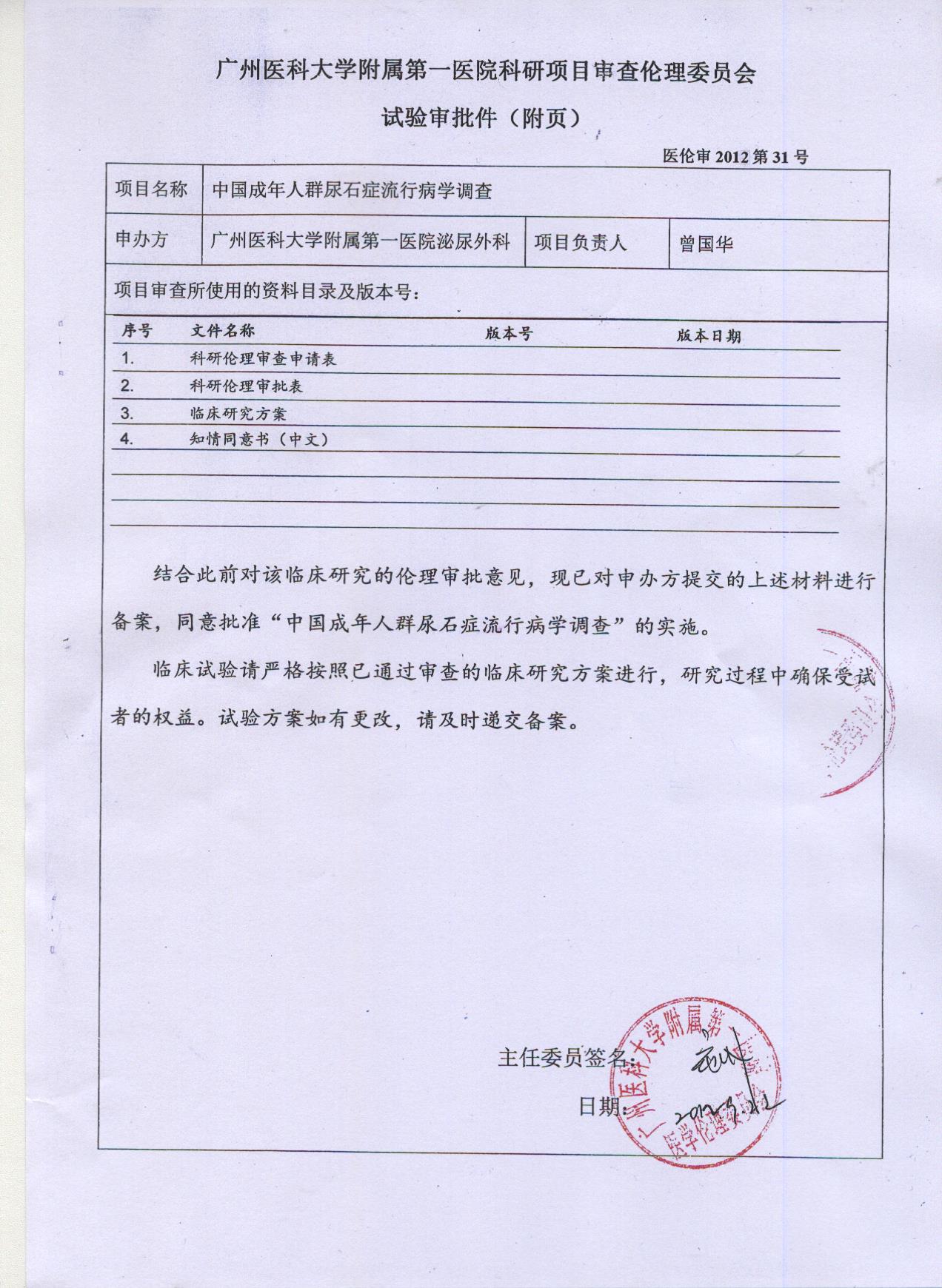
**
