# Supplementary material for: Prevalence of metabolic syndrome in China: An up-dated cross-sectional study
Source: PLoS One. 2018 Apr 18;13(4):e0196012. doi: 10.1371/journal.pone.0196012 (PMC5906019; doi:10.1371/journal.pone.0196012)
Supplement: S4 File — (DOCX) [file pone.0196012.s004.docx]

STROBE Statement—checklist of items that should be included in reports of observational studies

|  | Item No. | Recommendation | Page  No. | Relevant text from manuscript |
| --- | --- | --- | --- | --- |
| **Title and abstract** | 1 | (*a*) Indicate the study’s design with a commonly used term in the title or the abstract | 1 | Cross-sectional study in title. |
|  |  | (*b*) Provide in the abstract an informative and balanced summary of what was done and what was found | 2 | See the methods and finding in the abstract. |
| Introduction | | | |  |
| Background/rationale | 2 | Explain the scientific background and rationale for the investigation being reported | 3 | Metabolic syndrome is a worldwide public health problems which causing economic burden directly or indirectly. The prevalence of MS in China was 9.8% in 2005, and 10.5% in 2009. However, this situation might have changed since the rapid change of lifestyle and diet habit in China which is the biggest developing country in the world with a population more than 1.3 billion. Thereby it is worthy to survey the prevalence of China and preliminarily understand relevant risks or protect factors. |
| Objectives | 3 | State specific objectives, including any prespecified hypotheses | 3 | The aim of the present study was to provide reliable and up-to-date information on the prevalence and associated factors of MS among adults aged over 18 years in China. |
| Methods | | | |  |
| Study design | 4 | Present key elements of study design early in the paper | 3,4 | The subjects in this study were recruited from a cross-sectional survey, which was performed to estimate the prevalence of kidney stones among adults aged ≥18 years from May 2013 to July 2014 in China [the 5^th^ reference]. |
| Setting | 5 | Describe the setting, locations, and relevant dates, including periods of recruitment, exposure, follow-up, and data collection | 3-5 | See the section of subjects and data collection. |
| Participants | 6 | (*a*) *Cohort study*—Give the eligibility criteria, and the sources and methods of selection of participants. Describe methods of follow-up  *Case-control study*—Give the eligibility criteria, and the sources and methods of case ascertainment and control selection. Give the rationale for the choice of cases and controls  *Cross-sectional study*—Give the eligibility criteria, and the sources and methods of selection of participants | 3,4 | In the study, we selected the subjects by using a multistage, stratified sampling method which was stratified according to the traditional seven geographical regions of China (south, south central, southwest, east, north, northeast, and northwest). The sampling process was divided into four tiers according to the size of geographic area and population of the regions. In the first two tiers, we selected provinces from these seven regions in a non-randomized way, and selected cities and countries based on the degree of urbanization (cities vs villages). We selected streets (about 1000-3000 households) from the cities and villages (about 100-2 000 households) from the counties, and then selected households from streets and villages for recruiting subjects for randomized. All surveyed residents were aged 18 or older and had lived in their current region for six months or longer. |
|  |  | (*b*) *Cohort study*—For matched studies, give matching criteria and number of exposed and unexposed  *Case-control study*—For matched studies, give matching criteria and the number of controls per case |  |  |
| Variables | 7 | Clearly define all outcomes, exposures, predictors, potential confounders, and effect modifiers. Give diagnostic criteria, if applicable | 4-6 | See the section of data collection and assessment criteria. |
| Data sources/ measurement | 8* | For each variable of interest, give sources of data and details of methods of assessment (measurement). Describe comparability of assessment methods if there is more than one group | 4-6 | See the section of data collection and assessment criteria. |
| Bias | 9 | Describe any efforts to address potential sources of bias | 6 | All investigators involved in this study had completed a training program on the methodology and data acquisition procedures. Each of them had a procedure manual that detailed the administration of the questionnaires, operation of the urinary tract ultrasonography, measurements of the blood pressure and anthropometric indexes, along with collecting and handling the blood specimens. All the collected data were registered and treated with the Epidata software (version 3.1, Epidata Association, Denmark). |
| Study size | 10 | Explain how the study size was arrived at | 3,4 | The subjects in this study were recruited from a cross-sectional survey, which was performed to estimate the prevalence of kidney stones among adults aged ≥18 years from May 2013 to July 2014 in China [the 5th reference]. |

Continued on next page

| Quantitative variables | 11 | Explain how quantitative variables were handled in the analyses. If applicable, describe which groupings were chosen and why | 6,7 | See the section of statistical analysis. |
| --- | --- | --- | --- | --- |
| Statistical methods | 12 | (*a*) Describe all statistical methods, including those used to control for confounding | 6,7 | See the section of statistical analysis. |
|  |  | (*b*) Describe any methods used to examine subgroups and interactions | 6,7 | See the section of statistical analysis. |
|  |  | (*c*) Explain how missing data were addressed | 6,7 | See the section of statistical analysis. |
|  |  | (*d*) *Cohort study*—If applicable, explain how loss to follow-up was addressed  *Case-control study*—If applicable, explain how matching of cases and controls was addressed  *Cross-sectional study*—If applicable, describe analytical methods taking account of sampling strategy | 6,7 | See the section of statistical analysis. |
|  |  | (*e*) Describe any sensitivity analyses | N/A |  |
| Results | | | | |
| Participants | 13* | (a) Report numbers of individuals at each stage of study—eg numbers potentially eligible, examined for eligibility, confirmed eligible, included in the study, completing follow-up, and analysed | 7 | In total, 12 570 adults with an average age of 48.8±15.3 (18-96) were selected randomly and invited to participate in the study, and 2884 participants were unavailable to participate due to contact failure, refusal, out-of-town work, and serious disability, therefore, 9686 (77.1%) participants accepted the interview. Among these 9686 participants, 364 failed to complete the questionnaire and 12 got unusable blood samples, as a result, a total of 9310 individuals (3792 men and 5518 women) with an average age of 51.3±14.2 (18-96) years were included in the final analysis. The overall response rate was 74.1% (Fig 1). |
|  |  | (b) Give reasons for non-participation at each stage | 7 | See the first paragraph of results |
|  |  | (c) Consider use of a flow diagram | 7 | Figure 1 |
| Descriptive data | 14* | (a) Give characteristics of study participants (eg demographic, clinical, social) and information on exposures and potential confounders | 7,8 | The characteristics of participants were showed in Table 1. |
|  |  | (b) Indicate number of participants with missing data for each variable of interest | 7 | See the first paragraph of results |
|  |  | (c) *Cohort study*—Summarise follow-up time (eg, average and total amount) |  |  |
| Outcome data | 15* | *Cohort study*—Report numbers of outcome events or summary measures over time |  |  |
|  |  | *Case-control study—*Report numbers in each exposure category, or summary measures of exposure |  |  |
|  |  | *Cross-sectional study—*Report numbers of outcome events or summary measures | 8 | Totally, there were 1340 participants (576 men and 764 women) were defined with MS by duplication eliminating. |
| Main results | 16 | (*a*) Give unadjusted estimates and, if applicable, confounder-adjusted estimates and their precision (eg, 95% confidence interval). Make clear which confounders were adjusted for and why they were included | 7,8 | The age- and sex-adjusted prevalence of MS was estimated by the direct method using the known population distribution within China in 2010.  The overall prevalence of MS in China was 14.39% (95%CI: -3.75-32.53). The overall age adjusted prevalence of MS was 9.82% (95%CI: 9.03-10.61). |
|  |  | (*b*) Report category boundaries when continuous variables were categorized | 6,7 | See the section of statistical analysis. |
|  |  | (*c*) If relevant, consider translating estimates of relative risk into absolute risk for a meaningful time period | 9,10 | From the result of logistic regression analyses (Table 4), the factor of age, urolithiasis, hyperuricemia, coronary artery disease, thiazide drugs intake, family history of diabetes and hypertension were all significantly associated with an increased risk of metabolic syndrome (OR>1). In addition, education, vitamin D intake and family of urolithiasis seem to be protected factors (OR<1). |

Continued on next page

| Other analyses | 17 | Report other analyses done—eg analyses of subgroups and interactions, and sensitivity analyses | 7-10 | See the section of results. |
| --- | --- | --- | --- | --- |
| Discussion | | | | |
| Key results | 18 | Summarise key results with reference to study objectives | 10-15 | In this current cross-sectional study, the results indicate that 130.9 million (or 14·39%) adults aged over 18 years old in China have metabolic syndrome. |
| Limitations | 19 | Discuss limitations of the study, taking into account sources of potential bias or imprecision. Discuss both direction and magnitude of any potential bias | 15 | See the section of Limitation |
| Interpretation | 20 | Give a cautious overall interpretation of results considering objectives, limitations, multiplicity of analyses, results from similar studies, and other relevant evidence | 10-15 | See the section of Discussion and Limitation |
| Generalisability | 21 | Discuss the generalisability (external validity) of the study results | 15 | These findings indicated that metabolic syndrome have already become a major public health issue in China. Fortunately, we found that the prevalence of MS which used to be higher in urban [3] have become lower nowadays. It may result from the stronger awareness of avoiding metabolic diseases in urban resident in the past decade. In addition, this change means that with more reasonable nationwide strategies, the prevention and treatment of metabolic syndrome could possibly obtain a further progress. |
| Other information | |  | | |
| Funding | 22 | Give the source of funding and the role of the funders for the present study and, if applicable, for the original study on which the present article is based | 16 | See the section of Acknowledgments |

*Give information separately for cases and controls in case-control studies and, if applicable, for exposed and unexposed groups in cohort and cross-sectional studies.

**Note:** An Explanation and Elaboration article discusses each checklist item and gives methodological background and published examples of transparent reporting. The STROBE checklist is best used in conjunction with this article (freely available on the Web sites of PLoS Medicine at http://www.plosmedicine.org/, Annals of Internal Medicine at http://www.annals.org/, and Epidemiology at http://www.epidem.com/). Information on the STROBE Initiative is available at www.strobe-statement.org.
